# Costs Associated with Nontuberculous Mycobacteria Infection, Ontario, Canada, 2001–2012

**DOI:** 10.3201/eid2609.190524

**Published:** 2020-09

**Authors:** Lauren C. Ramsay, Emily Shing, John Wang, Theodore K. Marras, Jeffrey C. Kwong, Sarah K. Brode, Frances B. Jamieson, Beate Sander

**Affiliations:** University Health Network, Toronto, Ontario, Canada (L.C. Ramsay, T.K. Marras, J.C. Kwong, S.K. Brode, B. Sander);; University of Toronto, Toronto (L.C. Ramsay, T.K. Marras, J.C. Kwong, S.K. Brode, F.B. Jamieson, B. Sander);; Public Health Ontario, Toronto (E. Shing, J. Wang, J.C. Kwong, F.B. Jamieson, B. Sander);; ICES, Toronto (J. Wang, J.C. Kwong, S.K. Brode, B. Sander);; West Park Healthcare Centre, Toronto (S.K. Brode)

**Keywords:** tuberculosis and other mycobacteria, nontuberculous mycobacteria, cost, pulmonary disease, NTM pulmonary disease, NTM pulmonary isolation, Ontario, Canada, bacteria

## Abstract

To determine incidence-based healthcare costs attributable to nontuberculous mycobacterial (NTM) pulmonary disease (PD) and NTM pulmonary isolation (PI), from the healthcare payer perspective, we conducted a population-based matched cohort study in Ontario, Canada. We established cohorts of patients with incident NTM-PD and NTM-PI during 2001–2012 by using individually linked laboratory data and health administrative data, matched to unexposed persons from the general population. To estimate attributable costs for acute and long-term illness, we used a phase-of-care approach. Costs were stratified by age, sex, and healthcare resource, and reported in 2018 Canadian dollars (CAD) and US dollars (USD), standardized to 10 days. Costs were highest during the before-death phase (NTM-PD CAD $1,352 [USD $1,044]; NTM-PI CAD $731 [USD $565]). The cumulative mean attributable 1-year costs were CAD $14,953 (USD $11,541) for NTM-PD and CAD $8,729 (USD $6,737) for NTM-PI. Costs for patients with NTM-PD and NTM-PI were higher than those for unexposed persons.

Pulmonary disease (PD) caused by nontuberculous mycobacteria (NTM) is an emerging public health threat (*1*). As identification of persons with NTM-PD increases (*2*,*3*), information about the economic burden of NTM-PD can help decision makers set funding priorities. NTM-PD accounts for most NTM infections and poses particular challenges (*4*). One challenge is that NTM-PD often occurs in patients with preexisting conditions such as cystic fibrosis, lung cancer, and chronic obstructive pulmonary disease (*5*,*6*); these conditions affect the spectrum and severity of signs and symptoms (*7*). Treatment of NTM-PD is complex, typically involving use of >3 antimicrobial agents for >18 months (*5*). Eradication of the organism is challenging; a recent meta-analysis demonstrated a success rate of 60% for eradication of *Mycobacterium avium* complex PD (MAC-PD) (*8*). Furthermore, recurrence after completed treatment for MAC-PD is extremely common; recurrence rates are 30% at 14 months (*9*) and ≈50% at 4 years (*10*). During 1998–2010, the prevalence of NTM-PD in Ontario, Canada, increased; the 5-year prevalence increased from 29.3 (1998–2002) to 41.3 (2003–2007) cases/100,000 persons (*2*). 

The costs of NTM-PD in Canada are not well established; to the best of our knowledge, no studies have used population-based data to estimate associated costs. One study conducted in a tertiary care center in Toronto, Ontario, estimated the median monthly cost per NTM-PD patient to be approximately CAD $500; most of the costs were associated with medications (*11*). Although informative, that study considered only 1 clinic and did not account for potential long-term costs. Our objective with this study was to determine the costs associated with NTM-PD and with pulmonary isolation of NTM from patients without disease (NTM-PI), from the healthcare payer perspective, in Ontario, Canada, during 2001–2012. 

## Methods

### Study Design and Participants

We conducted a population-based matched cohort study to examine attributable costs of NTM-PD and NTM-PI from the healthcare payer perspective (Ontario Ministry of Health and Long-Term Care, Toronto, ON, Canada). Costs were identified by using provincial health administration data, including physician services, emergency department data, hospitalizations, prescription medications (for those >65 years of age), population data (e.g., census information and death records), and special collections (e.g., specific disease registries) (*12*). These data can be individually linked by using unique encoded identifiers at ICES (formerly the Institute for Clinical Evaluative Sciences), an independent, nonprofit research institute in Ontario, whose legal status under Ontario health information privacy law allows it to collect and analyze healthcare and demographic data without consent for health system evaluation and improvement (*12*). Descriptions of key ICES databases are described in more detail elsewhere (*13*–*15*). Our study was approved by the ethics review boards of Public Health Ontario and the University Health Network. All data analyses were conducted by using SAS version 9.4 (SAS Institute, https://www.sas.com).

We identified incident cases of NTM by using laboratory data from Public Health Ontario (https://www.publichealthontario.ca) for 2001–2012. NTM-PD was defined by the microbiological criteria of the American Thoracic Society/Infectious Diseases Society of America (ATS/IDSA) diagnostic guidelines: NTM isolation from >2 sputum samples (isolation of the same species within 2 years) or >1 positive sample from bronchoalveolar lavage or pleural fluid (*5*). NTM-PI was defined by NTM isolation from only 1 sputum sample. Persons were excluded if age, sex, or birth date data were not available; if they did not live in Ontario on the index date; if they were >100 years of age on the index date (i.e., the beginning of healthcare resource utilization related to NTM); if laboratory-confirmed *M. gordonae* had been isolated from them; or if laboratory-confirmed NTM had been isolated from them in the 3 years before the accrual period (January 1, 1998–December 31, 2000).

Because dates of disease onset were unknown, we adjusted index dates by using joinpoint (https://surveillance.cancer.gov/joinpoint) analysis on the cost curve (where a change in costs indicates change in healthcare use) and clinical judgement to estimate an onset of 30 days before laboratory confirmation of NTM. In the 30 days before laboratory confirmation, clinical judgement reconciled the increasing costs with the expectation of physician visits, possible hospitalizations, and a large number of clinical investigations.

Matched unexposed persons (who never had NTM-PD or NTM-PI) were drawn from the general population in the Registered Persons Database (https://datadictionary.ices.on.ca/Applications/DataDictionary/Library.aspx?Library=RPDB). We matched unexposed and exposed persons at a ratio of 3:1 by using a combination of hard-matching and propensity score matching without replacement (Tables 1, 2). We used a logistic regression model that regressed exposure status to calculate the propensity score based on the following covariates: rurality (using the Rurality Index of Ontario [*16*]), neighborhood income quintile, and underlying conditions 2 years before index date (using the Johns Hopkins Adjusted Clinical Groups System [*17*]). Persons were hard-matched by age, sex, and index date as well as within 0.2 SDs of the logit of the propensity score (*18*). To examine the effect of NTM-PD and NTM-PI on costs before death, we rematched each exposed person who died during the observation period (2001–2012) with 3 unexposed persons from the general population who also died during the same period, by using covariates assessed 180 days before death (Table 1).

**Table 1:** Variables used for matching persons exposed to nontuberculous mycobacteria to unexposed persons in study of costs associated with nontuberculous mycobacteria infection, Ontario, Canada, 2001–2012*

Variable	Index date	Death date
Baseline covariates–hard-matching		
Index date	± 30 d	NA
Death date	NA	± 90 d
Age	± 1 y	± 5 y
Sex	Exact	Exact
Propensity score variable		
Rurality Index of Ontario†	At index date	180 d before death
Neighborhood income quintile	At index date	180 d before death
Collapsed aggregated diagnosis groups‡	2 y before index date	

*Persons were hard-matched with regard to age, sex, and index date as well as within 0.2 SDs of the logit of the propensity score. To examine the effect of nontuberculous mycobacteria on costs before death, exposed persons who died during the observation period were rematched with 3 unexposed persons from the general population who also died during the same period, by use of covariates assessed 180 days before. NA, not applicable. †The Rurality Index of Ontario is a weighted function of community population/density, travel time to nearest basic referral center, and travel time to nearest advanced referral center (https://content.oma.org//wp-content/uploads/2008rio-fulltechnicalpaper.pdf). ‡The Johns Hopkins ACG system (https://www.hopkinsacg.org) groups comorbid diagnoses into clinical groups considering illness recurrence, severity, and resource use intensity.

**Table 2:** Baseline characteristics of exposed and unexposed persons after matching for NTM-PD and NTM-PI cohorts in study of costs associated with nontuberculous mycobacteria infection, Ontario, Canada, 2001–2012*

Characteristic	Exposed persons	Unexposed persons	Standardized difference
NTM-PD			
No. persons	7,243	21,729	
Mean age, y	66.1	66.1	0
Mean propensity score	6.6	6.6	0.01
Mean CADG score	5.5	5.5	0.02
Sex			
M	49.3	49.3	0
F	50.7	50.7	0
Income quintile			
1	26.8	24.2	0.06
2	21.3	21.0	0.01
3	18.1	17.7	0.01
4	16.7	18.0	0.03
5	17.2	19.1	0.05
Rurality			
Not rural	96.2	94.6	0.08
Rural	3.8	5.4	0.08
NTM-PI			
No. persons	8,393	25,179	
Mean age, y	61.0	61.0	0
Mean propensity score	6.7	6.7	0.01
Mean CADG score	5.1	5.1	0.02
Sex			
M	51.1	51.1	0
F	48.9	48.9	0
Income quintile			
1	31.0	28.9	0.05
2	23.3	22.1	0.03
3	16.9	17.2	0.01
4	15.1	16.3	0.03
5	13.8	15.5	0.05
Rurality			
Not rural	97.2	95.8	0.07
Rural	2.9	4.2	0.07

*Data are percentages unless otherwise indicated. CADG, collapsed aggregated diagnosis groups; NTM-PD, nontuberculous mycobacteria pulmonary disease; NTM-PI, nontuberculous mycobacteria pulmonary isolation.

### Outcomes

We evaluated deaths (10-day, 30-day, 90-day, 1-year), acute hospital admissions (within 5 and 30 days of index date), and hospital lengths of stay. We calculated costs by using person-level costing methods established by ICES (*14*). This method uses administrative data to calculate long-term costs of incident cases from an index date to a defined point in time (death or the end of an observation window). In this method, costs are inflated by using the healthcare-specific Consumer Price Index reported by Statistics Canada (*14*). The publicly funded healthcare service categories included were acute inpatient hospitalizations, emergency visits, same-day surgeries and other ambulatory treatments, inpatient rehabilitation, complex continuing care, long-term care, inpatient mental health, physician services, home care, eligible prescription medications, and devices (*14*).

### Cost Analyses

We calculated all costs in 2015 Canadian dollars (CAD) and present results in 2018 CAD and US dollars (USD). Costs were inflated from 2015 CAD by using the healthcare-specific Consumer Price Index (*19*) and converted to 2018 USD (1 CAD = 0.77 USD) (*20*). Using phase-of-care methods, we estimated NTM-PD– and NTM-PI–attributable healthcare costs for acute and long-term illness. We defined 3 phases (acute, continuing, and subsequent) by using joinpoint analysis (*21*) and clinical judgment. Clinical judgment supported the results of joinpoint analysis with consideration of typical patterns of physician visits, clinical investigations, treatment initiation, and illness duration. We divided the acute care (phase 1) into 2 parts: initial and subsequent. The remaining phases were continuing care (phase 2) and before death (phase 3). Phase 1 was estimated to last 150 days from the index date; the first 60 days were defined as initial care and the remaining 90 days as subsequent care. Phase 2 started 70 days before death. Observation time was divided into phases in order of final, initial, and subsequent care; the remaining observation time was allocated to continuing care. For example, if a person was observed for 400 days, the last 70 days were assigned to the before-death phase and the first 150 days to initial (60 days) and subsequent (90 days) care, cumulatively representing phase 1; the remaining 180 days were allocated to the continuing care phase.

We calculated phase-specific attributable costs as the mean difference between matched pairs, and we used bootstrapping to calculate the 95% CI of the mean difference (*22*). Costs were measured as 10-day intervals throughout the observation period. We also considered attributable costs by persons’ age category, sex, and healthcare spending category. We determined attributable mean 1-year costs by applying 10-day survival probabilities from the first year after diagnosis to the mean 10-day phase-specific costs as we described.

### Sensitivity Analysis

We conducted a sensitivity analysis by removing persons identified in the Ontario Cancer Registry (https://datadictionary.ices.on.ca/Applications/DataDictionary/Library.aspx?Library=OCR) and the Canadian Cystic Fibrosis Data Registry (https://datadictionary.ices.on.ca/Applications/DataDictionary/Library.aspx?Library=CFDR) as having any history of lung cancer or cystic fibrosis because of possible differences in expected complications and costs associated with these conditions. We performed cost analysis for the matched cohort without these persons and by using the same methods we described.

## Results

### Study Cohort

During 2001–2012, a total of 7,384 NTM-PD cases and 8,580 NTM-PI cases were identified and linked to administrative data (Table 3). The mean (± SD) age of NTM-PD patients was 66.1 (± 15.6) years and of NTM-PI patients was 61.1 (± 18.3) years. Of the NTM-PD patients, 3,732 (50.5%) were female, 316 (4.3%) lived in rural areas, and the mean collapsed aggregated diagnosis groups score was 5.5 (± 2.1). Of the NTM-PI patients, 4,158 (48.5%) were female, 285 (3.3%) lived in rural areas, and the mean collapsed aggregated diagnosis groups score was 5.1 (± 2.2).

**Table 3:** Baseline characteristics of patients with confirmed nontuberculous mycobacterial pulmonary disease and pulmonary isolation of nontuberculous mycobacteria, Ontario, Canada, 2001–2012*

Characteristic	NTM-PD	NTM-PI
Infected persons	7,384 (100)	8,580 (100)
Age category at index, y		
<5	<6 (<0.1)	<6 (<0.1)
5–24	<123 (<1.7)	<296 (<3.4)
25–44	633 (8.6)	1,466 (17.1)
45–54	799 (10.8)	1,201 (14.0)
55–64	1,272 (17.2)	1,398 (16.3)
65–74	1,953 (26.5)	1,834 (21.4)
75–84	2,078 (28.1)	1,775 (20.7)
>85	526 (7.1)	610 (7.1)
Sex		
F	3,732 (50.5)	4,158 (48.5)
M	3,652 (49.5)	4,422 (51.5)
Rural residence		
No	7,065 (95.7)	8,289 (96.7)
Yes	316 (4.3)	285 (3.3)
CADG distribution, score		
Mean ± SD	5.48 ± 2.1	5.08 ± 2.2
Median	6	5
Income quintile		
1 (lowest)	1,985 (27.0)	2,671 (31.3)
2	1,562 (21.2)	1,978 (23.2)
3	1,325 (18.0)	1,435 (16.8)
4	1,224 (16.6)	1,287 (15.1)
5 (highest)	1,260 (17.1)	1,165 (13.6)
Died	2,821 (38.2)	2,381 (27.8)

*Units are no. (%) unless otherwise indicated. CADG, collapsed aggregated diagnosis group.

Of the NTM-PD patients, 272 (3.7%) were admitted to a hospital within 5 days of the index date and 3,839 (52.0%) were admitted within 30 days. The mean (± SD) length of hospital stay was 11.1 (± 20.5) days for those admitted within 5 days and 10.7 (± 24.0) days for those admitted within 30 days. For these patients, the 90-day all-cause mortality rate was 6.0% (n = 444) and the 1-year rate was 13.9% (n = 1,024).

Of the NTM-PI patients, 241 (2.8%) were admitted to the hospital within 5 days and 2,294 (26.7%) within 30 days; mean (± SD) length of stay was 10.6 (± 22.9) days for those admitted within 5 days and 14.0 (28.5) days for those admitted within 30 days. For these patients, the 90-day all-cause mortality rate was 3.4% (n = 294) and the 1-year rate was 8.6% (n = 738).

We matched 7,243 (98.1%) of NTM-PD and 8,393 (97.8%) of NTM-PI patients to unexposed persons. All standardized differences were <0.1, indicating good balance (Table 2). We matched 3,116 (98.8%) of NTM-PD and 2,616 (97.8%) of NTM-PI patients who died and found that standardized differences were <0.1 for both groups.

### Cost Analysis

For NTM-PD and NTM-PI, the mean attributable costs over the first 3 phases declined to the lowest cost during the continuous care phase (CAD $236 [USD $182], 95% CI CAD $199–$272 [USD $154–$210] for NTM-PD; CAD $133 [USD $103], 95% CI CAD $111–$154 [USD $85–$119] for NTM-PI). Costs then increased to the highest costs in the before-death phase (CAD $1,352 [USD $1,044], 95% CI CAD $1,104–$1,601 [USD $852–$1,236] for NTM-PD; CAD $731 [USD $565], 95% CI $506–$958 [USD $390–$739] for NTM-PI) (Table 4). For NTM-PD and NTM-PI, hospitalizations accounted for the largest proportion of costs across all phases. In the initial infection phase, 67.9% (CAD $663 [USD $512]) of NTM-PD costs and 65.5% (CAD $415 [USD $320]) of NTM-PI costs were for hospitalization.

**Table 4:** Ten-day mean attributable costs by phase for nontuberculous mycobacterial pulmonary disease and nontuberculous mycobacterial pulmonary isolation, Ontario, 2001–2012*

Spending category (no. patients)	Cost, CAD		
	Exposed persons	Unexposed persons	Attributable (95% CI)
Nontuberculous mycobacterial pulmonary disease			
Initial infection (6,906)			
Total	1,209	232	977 (905–1,048)
Hospitalization	724	61	663 (603–723)
Emergency department	35	6	29 (27–31)
Drugs	60	37	23 (19–26)
Physicians	232	45	187 (177–196)
Other	158	83	75 (62–88)
Subsequent care (6,906)			
Total	713	218	494 (438–549)
Hospitalization	360	55	305 (258–352)
Emergency department	12	6	6 (5–7)
Drugs	67	37	30 (26–34)
Physicians	114	41	73 (68–79)
Other	160	80	80 (65–95)
Continuous care (6,489)			
Total	530	294	236 (199–272)
Hospitalization	196	75	120 (92–149)
Emergency department	13	8	5 (4–6)
Drugs	73	43	29 (26–33)
Physicians	77	45	33 (30–36)
Other	171	123	48 (34–61)
Before-death (2,835)			
Total	5,300	3,947	1,352 (1,104–1,601)
Hospitalization	3,757	2,492	1,265 (1,032–1,497)
Emergency department	111	108	2 (−2 to 6)
Drugs	110	105	5 (−3 to 14)
Physicians	573	417	156 (130–182)
Other	749	825	−76 (−128 to −24)
Nontuberculous mycobacterial pulmonary isolation			
Initial infection (8,171)			
Total	822	189	633 (591–676)
Hospitalization	464	49	415 (381–449)
Emergency department	32	5	27 (25–29)
Drugs	48	30	18 (15–20)
Physicians	158	38	120 (114–125)
Other	121	67	54 (42–67)
Subsequent care (8,171)			
Total	436	183	253 (222–284)
Hospitalization	178	45	133 (109–156)
Emergency department	11	5	6 (5–7)
Drugs	51	30	21 (17–24)
Physicians	80	37	43 (40–47)
Other	117	67	50 (39–62)
Continuous care (7,860)			
Total	387	253	133 (111–154)
Hospitalization	122	64	57 (42–72)
Emergency department	11	7	4 (3–5)
Drugs	57	36	22 (19–25)
Physicians	60	40	20 (18–22)
Other	136	106	29 (19–40)
Before death (2,374)			
Total	4,532	3,800	731 (506–958)
Hospitalization	3,088	2,351	736 (524–949)
Emergency department	124	108	17 (12–22)
Drugs	108	101	7 (−1 to 15)
Physicians	474	412	62 (40–83)
Other	738	827	−90 (−146 to −34)

*Costs presented in 2018 Canadian dollars (CAD). In 2018, $1 CAD = $0.77 US dollars.

For NTM-PD and NTM-PI patients, mean attributable hospitalization costs were highest before death (NTM-PD CAD $1,265 [USD $976], 95% CI CAD $1,033–$1,498 [USD $797–$1,156]; NTM-PI CAD $737 [USD $569], 95% CI CAD $524–$949 [USD $404–$732]) and second highest during the initial infection phase (NTM-PD CAD $663 [USD $512], 95% CI CAD $603–$723 [USD $465–$558]; NTM-PI CAD $415 [USD $321], 95% CI CAD $382–$450 [USD $294–$347]). Physician service costs were greatest during the initial infection stage, costing an average of CAD $187 (USD $144), 95% CI CAD $177–$196 (USD $137–$151) more than uninfected persons for NTM-PD and CAD $119 (USD $92), 95% CI CAD $114–$125 (USD $88–$97) more than uninfected persons for NTM-PI.

**Figure 1:**
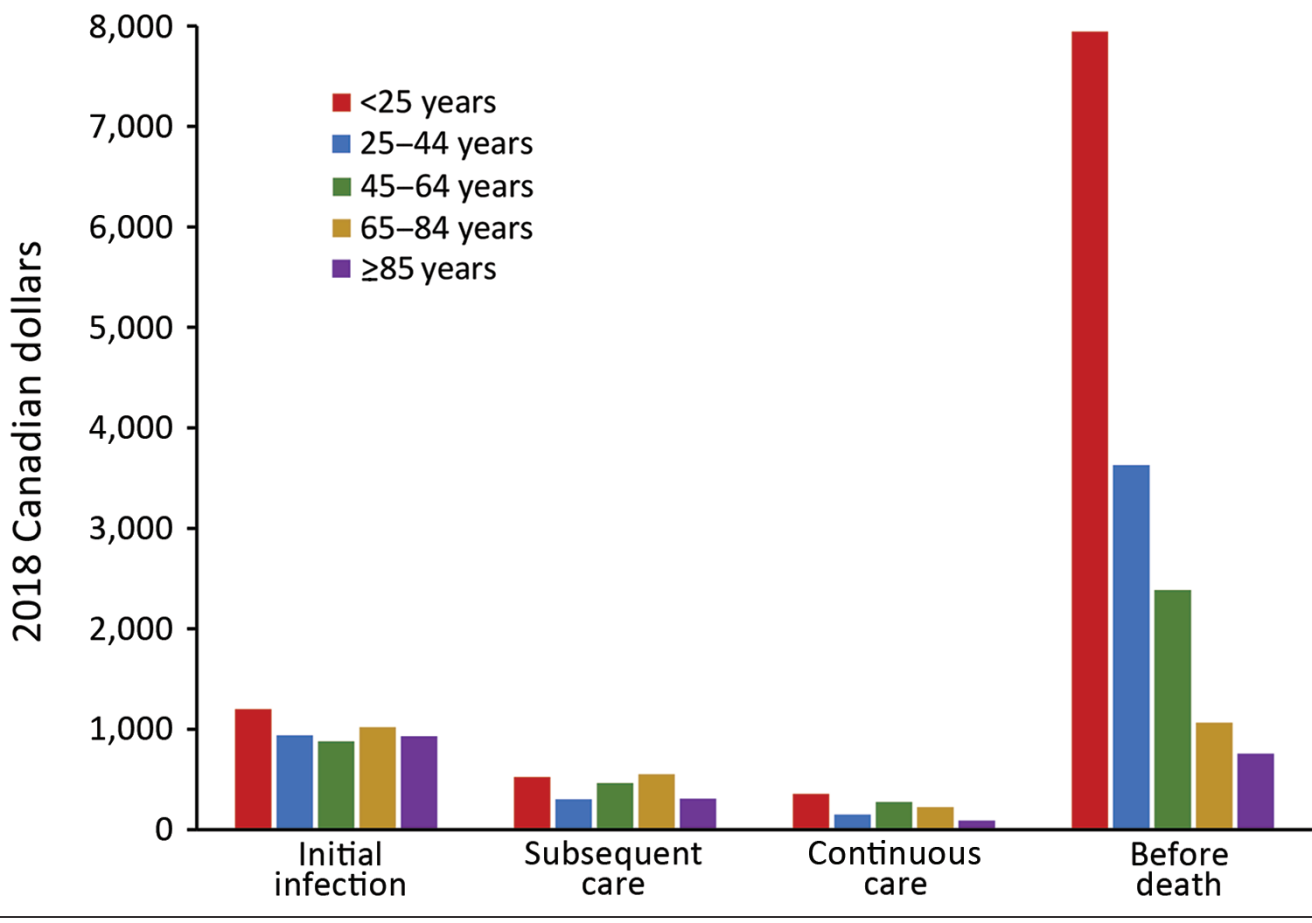
Ten-day mean attributable costs for nontuberculous mycobacterial pulmonary disease patients by phase, stratified by age, Ontario, Canada, 2001–2012. Number of patients per category: initial infection, 6,906; subsequent care, 6,906; continuous care, 6,489; before death, 2,835.

**Figure 2:**
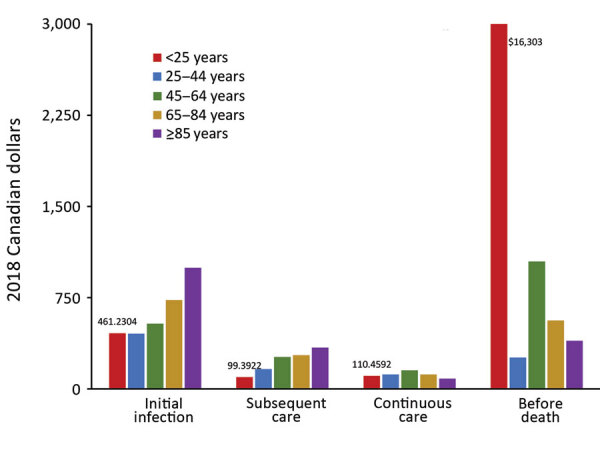
Ten-day mean attributable costs for nontuberculous mycobacterial pulmonary isolation patients by phase, stratified by age, Ontario, Canada, 2001–2012. Number of patients per category: initial infection, 8,171; subsequent care, 8,171; continuous care, 7,860; before death, 2,374.

For NTM-PD patients, costs were greatest during the before-death phase and the initial infection phase (Figure 1). The highest costs before death were found for patients in the <25 years age group (CAD $7,952 [USD $6,138], 95% CI CAD $3,840–$19,744 [USD –$2,963 to $15,238]; n= 7) and declined in each subsequent age group; the lowest costs before death were found for patients >85 years of age (CAD $762 [USD $588], 95% CI CAD $379–$1,145 [USD $293–$883]; n = 778). Similarly, the highest costs before death for NTM-PI patients were for those <25 years of age (CAD $16,303 [USD $12,583], 95% CI CAD $16,161–$16,446 [USD $12,473–$12,692], n<6); however, the lowest attributable costs were for those 25–44 years of age (CAD $261 [USD $202], 95% CI CAD –$2,519 to $3,041 [USD –$1,944 to $2,347]; n = 57), followed by declining costs in each of the subsequent age categories: 45–64 years (n = 351), 65–84 years (n = 1,440), and >85 years (n = 822) (Figure 2).

**Figure 3:**
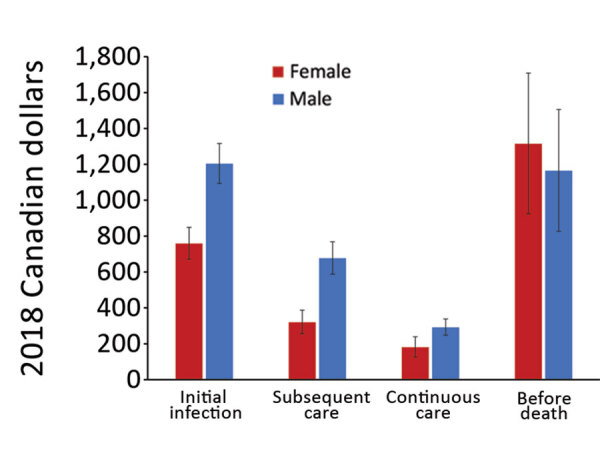
Ten-day mean attributable costs for nontuberculous mycobacterial pulmonary disease patients by phase, stratified by sex, Ontario, Canada, 2001–2012. Number of patients per category: initial infection, 6,906; subsequent care, 6,906; continuous care, 6,489; before death, 2,835. Error bars indicate 95% CIs.

**Figure 4:**
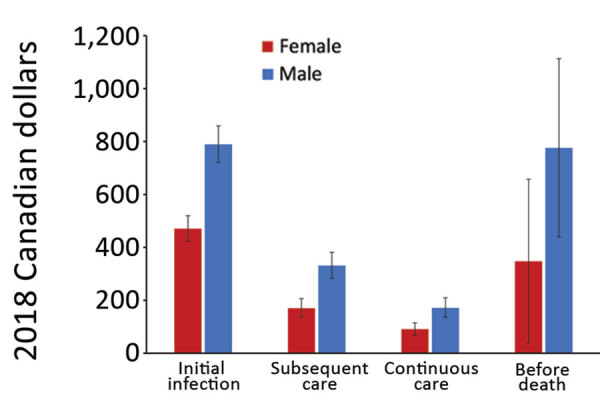
Ten-day mean attributable costs for nontuberculous mycobacterial pulmonary isolation patients by phase, stratified by sex, Ontario, Canada, 2001–2012. Number of patients per category: initial infection, 8,171; subsequent care, 8,171; continuous care, 7,860; before death, 2,374. Error bars indicate 95% CIs.

Overall, mean attributable costs were higher for male than for female NTM-PD patients in all phases except before death, when attributable costs were higher for female than male patients (Figure 3). In the before-death phase, NTM-PD female and male attributable costs were CAD $1,316 (USD $1,016), 95% CI CAD $925–$1,709 (USD $714–$1,319) for female patients and CAD $1,166 (USD $900), 95% CI CAD $826–$1,505 (USD $638–$1,162) for male patients. For NTM-PI patients during all phases, mean attributable costs were higher for male than for female patients (Figure 4). The mean attributable cumulative 1-year costs adjusted for survival were CAD $14,953 (USD $11,541) per NTM-PD patient and $CAD 8,729 (USD $6,737) per NTM-PI patient.

### Sensitivity Analysis

After removing from analysis persons with a history of lung cancer or cystic fibrosis, we matched 6,461 NTM-PD and 7,887 NTM-PI patients to unexposed persons. For NTM-PD, total mean attributable 10-day costs per patient were CAD $951 (USD $734), 95% CI CAD $875–$1,027 (USD $675–$792) for initial infection; CAD $428 (USD $330), 95% CI CAD $370–$485 (USD $286–$374) for subsequent care; CAD $190 (USD $146), 95% CI CAD $153–$227 (USD $118–$175) for continuous care; and CAD $1,479 (USD $1,141), 95% CI CAD $1,176–$1,780 (USD $908–$1,374) for before-death phases (Table 5). For the same phases, the total mean attributable costs for NTM-PI patients were CAD $614 (USD $474), 95% CI CAD $570–$659 (USD $440–$508); CAD $220 (USD $170), 95% CI CAD $189–$253 (USD $146–$195); CAD $108 (USD $83), 95% CI CAD $85–$130 (USD $66–$100); and CAD $850 (USD $656), 95% CI CAD $589–$1,110 (USD $455–$857). For NTM-PD patients, the attributable costs in the sensitivity analysis were significantly lower during the subsequent care and continuous care phases. For NTM-PI patients, the costs were significantly lower during the continuous care phase only.

**Table 5:** Sensitivity analysis 10-d mean attributable costs by phase for nontuberculous mycobacterial pulmonary disease and nontuberculous mycobacterial pulmonary isolation, Ontario, Canada, 2001–2012*

Phase (no. patients)	Cost, CAD		
	Exposed persons	Unexposed persons	Attributable (95% CI)
Nontuberculous mycobacterial pulmonary disease			
Mean cost by phase			
Initial infection (6,187)	1,189	238	951 (875–1,027)
Subsequent care (6,187)	653	227	428 (370–485)
Continuous care (5,907)	493	304	190 (153–227)
Before death (2,204)	5,662	4,183	1,479 (1,176–1,781)
Nontuberculous mycobacterial pulmonary isolation			
Mean cost by phase			
Initial infection (7,693)	807	193	614 (570–659)
Subsequent care (7,693)	412	192	220 (189–253)
Continuous care (7,471)	370	261	108 (85–130)
Before death (1,963)	4,692	3,843	850 (589–1,110)

*Analysis excluded excluding persons with a history of lung cancer or cystic fibrosis. CAD, 2018 Canadian dollars. In 2018, $1 CAD in 2018 = $0.77 US dollars.

## Discussion

In this study, we found higher attributable healthcare costs to be associated with persons with NTM-PD or NTM-PI compared with persons without NTM. The highest costs were associated with hospitalizations, particularly during the initial infection and before-death phases; overall costs were markedly lower during the subsequent care and continuous care phases. Hospital admissions within 30 days of index date were more common among NTM-PD (52%) than NTM-PI (27%) patients. Incident cases in Ontario during our observation period were an average of 225 cases of NTM-PD and 191 cases of NTM-PI. On the basis of the cumulative 1-year costs, this finding would translate into an estimated total annual healthcare cost of CAD $3,369,765 (USD $2,600,730) for NTM-PD and CAD $1,667,863 (USD $1,287,229) for NTM-PI.

For all phases, 10-day mean attributable costs were higher for patients with NTM-PD than for those with NTM-PI. However, healthcare costs were significantly higher for patients with NTM-PI than for persons without NTM infection. Although 1 positive sputum sample may be clinically insignificant for some persons, for others it might represent the single isolation of a chronically present organism. It is therefore possible that some persons with NTM-PI had true disease, which may explain the higher healthcare costs. Alternatively, the higher healthcare costs may relate to a non-NTM lung condition that prompted specimen collection for culture, whereby the positive NTM culture was a nonsignificant bystander. Unfortunately, we do not have data on negative cultures to explore this further.

Overall, mean attributable costs for some phases were somewhat lower in the sensitivity analysis after we removed from analysis persons with a history of lung cancer or cystic fibrosis, which may result from expected increased costs for patients with related conditions. The lack of larger differences in attributable costs between these analyses may be explained by effective matching that used comorbidity scores that may have already accounted for these conditions in the cohort used for the primary analysis.

This study has limitations. Secondary use of health administrative data are prone to errors that could result in misclassification bias. Regarding the diagnosis of NTM-PD, we assumed that all patients who fulfilled the microbiological criteria of the ATS/IDSA guidelines truly had cases of NTM-PD. This assumption is highly accurate (positive predictive value 70%–100%), but some patients are invariably incorrectly classified as having NTM-PD (*23*–*26*). However, the finding that attributable healthcare costs for patients with NTM-PI were comparably high supports the finding of high costs associated with pulmonary NTM infection. In addition, the retrospective use of administrative data is limited by the variables that are available. Because this research was conducted from the healthcare payer perspective, the results capture only direct healthcare costs of NTM-PD and NTM-PI, not societal or indirect costs, which includes not capturing all medication costs associated with NTM because these costs are captured only for those with publicly funded medication coverage (i.e., adults >65 years of age and those receiving social assistance).

This study was strengthened by using both hard-matching and propensity score matching to reduce bias between unexposed and exposed persons. By matching on major covariates, we reduced the potential confounding by these covariates, allowing for a more robust estimate of NTM-PD and NTM-PI attributable costs. This study was further strengthened by using Ontario health administrative data, which contain extensive data on Ontario population healthcare use. Ontario is the most populous province in Canada (≈40% of the population) and is fairly representative of the population of Canada (*27*). In addition, the Public Health Ontario laboratory processes ≈95% of NTM isolates for the province (*28*), providing almost complete capture of microbiologically defined incident NTM-PD and NTM-PI cases in Ontario.

Previous studies have estimated the economic costs of NTM-PD in Canada and elsewhere. One group focused on the direct costs of NTM without assessing total healthcare costs. A retrospective study of 91 patients with NTM-PD treated at a clinic in Ontario reported that CAD $500/month was associated with NTM treatment, including costs of medications and their administration, physicians’ fees, and diagnostic tests (*11*). That study, however, was unable to study all healthcare costs and was limited to 1 clinic, introducing substantial bias. One study in the United States estimated the cost of antibiotic treatment (but excluded costs of administration) for NTM-PD patients during 2004–2005 and found that among 27 eligible patients, the median monthly treatment cost was USD $481 (*29*). Comparing the results of this study to the results from our analysis is difficult because not all medication costs are captured in Ontario’s health administrative data and because of differences in costs associated with healthcare systems in Canada and the United States. Another study conducted in the United States, which sought to estimate the direct costs of investigating and managing NTM-PD, used Medicare beneficiary data and costs from the literature to determine that medication costs made up most of NTM direct healthcare costs (*30*). Another study of direct NTM costs focused on patients with refractory MAC-PD. That study used a physician survey method and found that in Canada, average annual direct medical costs were CAD $16,200 and also presented data for Germany, France, and the United Kingdom (costs 9,700–17,900 Euros) (*31*). Although clear that the costs associated with refractory MAC-PD were high, the biased population (refractory disease) makes comparison with our study results difficult. Furthermore, the above-mentioned studies ignored total healthcare costs, which are undoubtedly influenced by the effects of NTM-PD on other diseases.

A second group of studies included all healthcare costs associated with NTM-PD. In a study performed in a managed care population in the United States, total healthcare costs for NTM-PD patients exceeded those for controls by USD $44,070 in the first year after diagnosis and $19,124 in the second (index date) (*32*). Limitations of that study included the lack of phase-of-illness modeling and the use of hard-matching by age and sex and statistical adjustment for underlying conditions. The results of propensity score matched analyses led to attenuation of the differences, which were still substantial. The comparison between the sampling of a US population enrolled in certain managed care programs and a population-based Ontario sample is difficult, but both studies identified a substantial increase in cost among patients with pulmonary NTM. A study in Germany investigated total healthcare costs among patients with incident NTM-PD compared with controls matched by age, sex, and Charlson Comorbidity Index category, identifying patients from a large and likely representative national-level database (*33*). Studied costs were incurred within the 3 years after NTM diagnosis; mean total healthcare expenditures for NTM-PD patients were ≈4 times those of controls (39,599 vs. 10,006 Euros) (*33*). The comparison was limited by probable inadequate matching, in that Charlson Comorbidity Index category probably provides relatively coarse discrimination among patients with varying levels of illness severity.

The qualitative results of our study may be transferable to jurisdictions with similar healthcare systems (i.e., publicly funded) and with similar population health profiles, but the magnitude of cost associated with NTM undoubtedly varies according to system-specific costs. NTM-PD and NTM-PI are responsible for substantial economic burden in Ontario. These results can be used in future economic evaluations to inform policy making on prevention, screening, and treatment options.
